# The chicken gut microbiome in conventional and alternative production systems

**DOI:** 10.1186/s40104-025-01293-8

**Published:** 2025-11-19

**Authors:** Yu-Chieh Cheng, Margret Krieger, Anna-Maria Korves, Amélia Camarinha-Silva

**Affiliations:** 1 University of Hohenheim, Institute of Animal Science, Stuttgart, Germany; 2 HoLMiR – Hohenheim Center for Livestock Microbiome Research, Stuttgart, Germany; 3 Department of Animal Nutrition and Animal Health, University of Kassel, Witzenhausen, Germany; 4 Department Biological Safety, German Federal Institute for Risk Assessment, Berlin, Germany

**Keywords:** Antimicrobial resistance, Chicken, Gut microbiome, Organic, Production systems

## Abstract

The poultry gut microbiome plays a key role in nutrient digestion, immune function, and overall health. Differences among various farming systems, including conventional, antibiotic-free, free-range, and organic systems, influence microbial composition and function through variations in diet, genetic selection, environmental exposure, and antibiotic use. Conventional systems typically rely on formulated diets and controlled housing conditions, often with routine antimicrobial use. In contrast, organic systems emphasize natural feed ingredients, including roughage, outdoor access, and strict limitations on the use of antibiotics. These divergent practices shape the gut microbiota differently, with organic systems generally associated with greater exposure to environmental microbes and, consequently, greater microbial diversity. However, the implications of this increased diversity for poultry health and performance are complex, as organic systems may also carry a higher risk of pathogen exposure. This review summarizes current findings on the chicken gut microbiome across conventional and alternative production systems (antibiotic-free, free-range, and organic), focusing on microbial diversity, functional potential, and disease resilience. The need for standardized methodologies and consistent nomenclature in microbiome research is also discussed to improve comparability across studies. Understanding how production systems influence the gut microbiota is essential for improving poultry health and productivity while addressing challenges related to antimicrobial resistance and sustainable farming practices.

## Introduction

Chicken is the most widely consumed meat worldwide, with poultry production continuing to increase worldwide. In 2023, approximately 76.2 billion chickens were raised for meat, representing an 82% increase compared with 2001 [1, 2]. To meet this growing demand, chicken farming has expanded significantly worldwide, with conventional production systems becoming the predominant method for chicken meat production. These systems are characterized by high stocking densities, genetic selection for rapid growth, and optimized feeding strategies and management practices aimed at maximizing yields and productivity [3–5]. However, such intensive rearing systems have raised concerns regarding animal welfare, the widespread use of antibiotics, and environmental sustainability. Issues such as leg deformities, elevated stress levels, and increased fear responses have been associated with rapid growth rates and intensive housing conditions [6]. Moreover, the extensive use of growth-promoting antibiotics (GPAs) in these systems has contributed to the emergence of antimicrobial resistance (AMR), which poses a significant threat to both animal and human health [7, 8]. In regions where GPAs have been banned (European Union (EU) since 2006, following the United States), conventional farms may operate with therapeutic-only antibiotic use or adopt entirely antibiotic-free programs while maintaining intensive production characteristics [9]. In contrast, conventional operations in regions without GPA restrictions may continue antibiotic use. Thus, modern conventional production represents a spectrum of practices unified by intensive management and genetic selection for performance rather than by antibiotic use alone. Environmental sustainability is also a concern because of the high energy demands and greenhouse gas emissions associated with feed production and transportation, as well as the eutrophication and acidification potential linked to manure management. In response to growing consumer awareness regarding food safety, sustainability, and animal welfare, the demand for alternative poultry production systems has increased [10, 11]. Consequently, alternative rearing systems such as the free-range system and organic farming have gained popularity as potential solutions to welfare concerns in broiler chickens [12–14]. The free-range system provides outdoor access, allowing chickens more freedom of movement and the opportunity to express natural behaviors, which are essential for improving animal welfare. In recognition of these benefits, many countries and regulatory bodies have implemented policies supporting such systems, reflecting the broader shift in consumer preferences toward ethically produced poultry. As a result, free-range practices are increasingly influencing industry standards and shaping poultry production norms [15]. In addition, antibiotic-free production systems have emerged in response to concerns about antibiotic residues and resistant bacteria in poultry products and the risk of AMR transmission from animals to humans [16]. Organic poultry farming further extends these principles by implementing strict regulations related to animal welfare, feed composition, and the use of antibiotics. Organic systems typically involve slow-growing chicken genotypes, relatively low stocking densities, and mandatory outdoor access, all of which promote the expression of natural behaviors and support bird health. Additionally, organic farming also requires certified organic feed and enforces stricter limitations on antibiotic use than conventional farming [15].

**Fig. 1:**
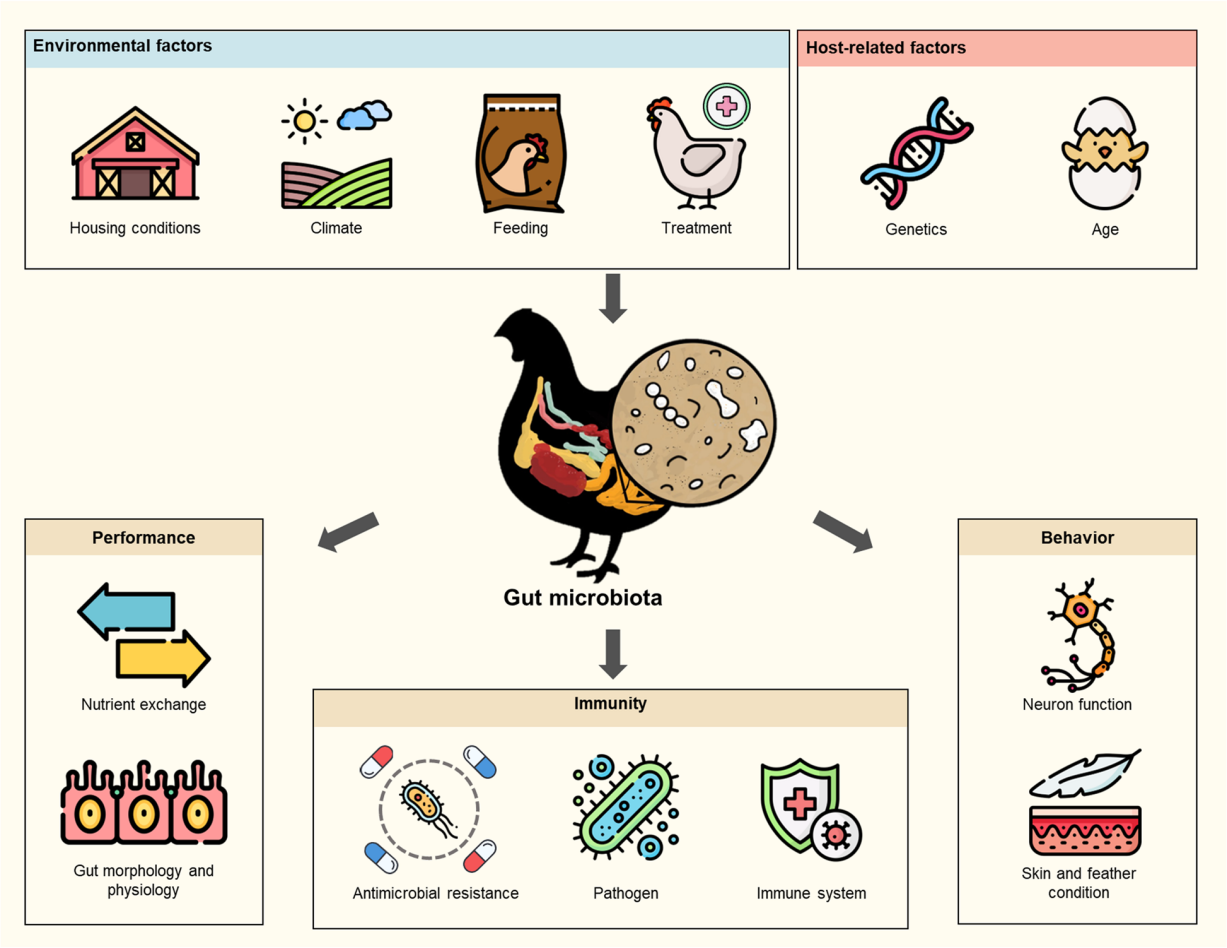
Host and environmental factors affecting chicken gut microbiota and health

The gut microbiota plays a crucial role in host physiology, influencing digestion, immune function, and overall health. It acts as an interface between the external environment and the host, modulating gut morphology, immune responses, and even behavior [17–19]. The composition and diversity of the gut microbiota are shaped by a combination of host-related and environmental factors (Fig. 1). Key environmental factors include farm location, climate, housing conditions, diet, medication use, and hygiene practices. Host-related factors such as age, breed, sex, and gastrointestinal tract (GIT) region also have an influence [20, 21]. Given the increasing recognition of microbiota-mediated effects on health, understanding the gut microbiome across different poultry production systems has become a critical area of research. By comparing conventional, antibiotic-free, free-range, and organic production systems, this review aims to elucidate how farming practices influence microbiota composition, with implications for poultry health, welfare, and sustainable production.

## The chicken gut microbiota

In recent years, the chicken gut microbiota has received increasing attention because of its key role in poultry health, nutrition, and productivity. Understanding the complex interactions within the gut microbiome is essential not only for optimizing poultry production and ensuring food safety but also for enhancing host performance and mitigating the transmission of zoonotic pathogens. The composition of the gut microbiota varies significantly across different segments of the GIT and is influenced by factors such as age, genetics, and environmental conditions. Despite this variability, a core microbiota persists in the chicken gut, comprising commensal bacteria that support gut health and maintain homeostasis [22, 23]. The predominant bacterial phyla include Bacillota (formerly Firmicutes, according to the updated nomenclature by the International Committee on Systematics of Prokaryotes) [24], Bacteroidota (formerly Bacteroidetes), Actinomycetota (formerly Actinobacteria), and Pseudomonadota (formerly Proteobacteria). Bacillota and Pseudomonadota dominate the cecal microbiota in young chickens, while Bacteroidota abundance increases during the later stages of life [23, 25].

In addition to their role in digestion and host metabolism, the gut microbiota also harbors several human pathogens of concern for human health. Among those, *Campylobacter* and *Salmonella* are highly relevant due to their zoonotic potential. *Escherichia coli* and certain members of the *Clostridium* genus, although considered part of the normal flora, can also act as opportunistic pathogens under specific conditions [23].

The microbial composition varies between intestinal segments due to differences in nutrient availability, retention time, and environmental conditions, which influence bacterial colonization and community structure [20]. Bacteria that produce lactic acid such as Enterobacteriaceae, *Lactobacillus*, *Bifidobacterium*, and *Klebsiella* are particularly abundant in the upper digestive tract of adult chickens, although other microbial species are also present [22, 26]. In contrast, microbial composition and complexity increase substantially in distal sections of the GIT, particularly in the cecum and colon [22, 27]. In the cecum, genera such as *Alistipes*, *Blautia*, *Ruminiclostridium*, and *Ruminococcus torques* are enriched [22]. The colonic microbiota is more variable and may resemble either the ileal or cecal communities, reflecting the anatomical and functional characteristics of the chicken digestive system [28, 29].

Research has revealed significant variability in gut microbiota composition, which is influenced by external environmental factors and host-specific characteristics. This complexity underscores the need for a comprehensive understanding of the factors that influence gut microbiota dynamics, particularly those related to different production systems. The following sections explore how various factors, including rearing systems, influence gut microbiota diversity and composition, thereby contributing to a deeper understanding of the interplay between microbial communities and host health. Notably, much of the literature cited in this review focuses specifically on caeca microbiota. The cecum is one of the most studied GIT segments in poultry, as it represents a crucial site for microbial fermentation and, to a lesser extent, nutrient absorption.

## Host and environmental modulators of the gut microbiota in chickens

Advances in high-throughput sequencing technologies have significantly improved our understanding of the microbial composition and functional potential of the chicken gut microbiota [19]. These developments have revealed that gut microbiota is a dynamic ecosystem that adapts continuously throughout bird’s life. Both host-related and environmental factors influence microbial colonization, community structure, and functional capabilities while shaping host metabolism, immunity, and overall health [20, 30]. This chapter explores the impact of age, genetics, and seasonal factors on the gut microbiota and host–microbe interactions in chickens.

### Age-related changes in gut microbiota

The composition of the intestinal microbiota in chickens changes significantly throughout their lifespan [20, 25, 27, 31, 32]. Microbial transmission begins even before hatching through maternal transfer via the oviduct and eggshell pores [33–36]. However, vertical transmission is likely limited in conventional production systems, which involve the separation of chicks from adults and egg disinfection practices [37]. After hatching, chicks acquire microbes from their environment, including hatcheries, transport systems, and farms [36]. Microbial diversity in the cecum increases with age, accompanied by distinct shifts in community structure. However, feces exhibit the highest microbial diversity immediately after hatching, whereas no significant differences were observed in the duodenal and ileal microbiota at this stage [27, 38, 39]. The microbiota exhibits a distinct structure immediately after hatching, being primarily dominated by rapidly colonizing or vertically transmitted microbes [40–42]. As the chickens age, the gut is initially colonized by fast-growing taxa, which are subsequently replaced by slower-growing species, ultimately leading to a more stable microbial community [41]. The microbiota remains relatively stable during the rapid skeletal development phase and then undergoes significant changes during the period of accelerated weight gain. These patterns indicate that chickens' gut microbiota composition evolves in a stage-specific manner during development [27, 40, 42]. The early ileal microbiome is generally dominated by members of the Enterobacteriaceae or Clostridiaceae families; however, in some cases, high relative abundances of other families such as Streptococcaceae and Enterococcaceae have also been reported [39, 41, 43, 44]. Lactobacillaceae is typically present at low relative abundance during this early stage. Studies have shown that age-related decreases in the relative abundance of Enterococcaceae [31, 45] and Lachnospiraceae [31] families in ileal digesta. Early colonization by Clostridiaceae is gradually replaced by Lactobacillaceae, with *Lactobacillus* and *Enterococcus* emerging as the dominant genera in the ileal microbiota [39, 46]. At the genus level, *Escherichia/Shigella* are the most common representatives of Enterobacteriaceae, whereas Clostridiaceae is most frequently assigned to *Clostridium *sensu stricto* 1* [36, 39, 46]. In the cecal digesta, Enterobacteriaceae and Clostridiaceae are dominant immediately after hatching [25, 40, 42, 44]. Oscillospiraceae (formerly Ruminococcaceae) and Lachnospiraceae are also prevalent in early life but show a gradual decline with age [25, 27, 31, 44, 45]. Certain genera display age-dependent trends. For example, *Clostridium *sensu stricto* 1*, is more abundant during the posthatching stage. Although it is considered a pioneer colonizer of the cecum, its abundance declines rapidly as the chickens' GIT matures. Researchers speculate that the high abundance of *Clostridium *sensu stricto* 1* in day-old chicks may be attributed to immune immaturity and increased susceptibility to environmental colonizers [27, 36, 41, 42, 46, 47]. Other genera, such as *Lactobacillus* and *Escherichia/Shigella*, also undergo dynamic age-related shifts in abundance [27, 48]. *Lactobacillus* is initially present in low abundance but increases over time [27, 49]. Its presence is associated with weight gain [50] and feed efficiency [51], suggesting a regulatory role in gut health [52]. In contrast, *Escherichia/Shigella* are negatively associated with growth performance and fat digestibility in chickens [30]. Age-related changes in the gut microbiota indicate that microbial colonization is a dynamic process, influenced by factors such as diet, immune responses, and interactions with the host and other microbes [42, 43, 53].

### Genetic influence on gut microbiota

Host genetics significantly influence the structure and function of chicken gut microbiota. For example, Schokker et al. [54] demonstrated that the genetic background affects both gut microbiota colonization and functional activities. They reported distinct differences in microbial composition between two broiler lineages, despite comparable overall diversity. In early life, differences in jejunal gene expression, particularly those related to immune function, cell proliferation, and epithelial barrier integrity, were observed, resulting in unique gut environments that favor different bacterial populations [54].

Fast-growing chickens selected for rapid weight gain exhibit distinct microbial signatures, characterized by lower abundances of *Bacteroides* and *Lactobacillus*, and higher levels of *Cloacibacillus* populations [55]. These compositional differences translate into functional consequences, with fast-growing broilers showing increased susceptibility to metabolic stress and cardiovascular illness. In contrast, slow-growing, dual-purpose birds show upregulation of immune-related pathways and greater microbial contributions to disease resistance, as predicted by gut microbiome functional profiling [55].

In addition to the growth rate, selection for specific metabolic traits also shapes the microbiome. Compared with fat-line chickens, lean-line chickens harbor greater *Bacteroides* abundance [56]. Functional annotation revealed significant differences in energy metabolism pathways, particularly among short-chain fatty acid-producing bacteria and potential pathogens such as *Enterococcus*. Distinct profiles in microbial pathways related to obesity, adiposity, and energy balance regulation were observed between the two lines [56].

The contrast between commercial and heritage breeds further illustrates the role of host genetics. Díaz-Sánchez et al. [57] reported that genetic differences in microbiota composition, particularly in the phylum Bacteroidota, between two lines became more pronounced later in life [57]. Similarly, Emami et al. [58] found that genetic selection reshaped gut microbiome functionality, with modern broilers enriched in carbohydrate and lipid metabolism pathways, while heritage lines harbored more diverse amino acid and vitamin biosynthesis genes. The study identified *Lactobacillus* and *Faecalibacterium* as key contributors to these metabolic differences, emphasizing the joint influence of host genetics and feeding physiology on microbial community function [58].

When comparing high-performing commercial broiler lines, such as the Cobb line and the Legacy line, which are not subject to selective breeding [59], *Akkermansia* is abundant in the Cobb line but absent in the Legacy breed. This variation likely reflects physiological changes in the mucosa structure, as *Akkermansia* plays a role in mucin degradation and is associated with host metabolic health [60]. In another study, commercial Cobb broilers presented higher Actinomycetota abundance in the jejunum, whereas indigenous Omani chickens presented more Clostridia [61]. These differences likely reflect structural variations in the intestine, such as differences in villus length and crypt depth, which affect absorptive capacity, brush border enzyme secretion, and nutrient uptake [59, 61, 62].

Modern broilers, characterized by high feed intake and rapid digesta passage, tend to favor microbiota enriched in pathways for carbohydrate and lipid metabolism. In contrast, legacy lines demonstrate greater reliance on microbial amino acid and vitamin metabolism pathways, reflecting adaptations to different nutritional strategies [58]. Selective breeding has altered important physiological traits in chickens, including growth performance, gastrointestinal function, absorption ability, hormone secretion, and immune responsiveness. These host-level changes, in turn, modify the intestinal environment and drive differences in microbial community composition, diversity, and function [57, 61].

### Seasonal variability in gut microbiota

Seasonal variation significantly influences the composition and diversity of the chicken gut microbiota, particularly in the cecum. Compared with those that hatched in spring or summer, chickens that hatched in winter presented lower taxonomic richness and fewer bacterial genera. These seasonal effects are most pronounced during early life but persist throughout the rearing period [32, 63]. The cecal microbiota in summer was more diverse than that in fall, whereas in the ileum, seasonal changes affected species evenness rather than richness. Some researchers have even reported that season is a stronger determinant of cecal microbial composition than antibiotic treatment [32]. Temperature is one of the most important seasonal variables, and heat stress is considered the greatest challenge for rearing chickens in summer [64]. Gut microbiota function and community composition are impacted by heat stress [58, 64–66]. Liu et al. [64] suggested that exposure to heat stress increased the levels of proinflammatory cytokines in the jejunum, which are likely linked to dysbiosis, particularly the decreased amounts of *Bdellovibrio* and *Ruminococcus*. Heat stress increased Lactobacillales and *Faecalibacterium* but decreased Enterobacteriales in chick ileal contents [66]. Compared with those in the control group, heat stress increased the relative abundances of Bacillota, Mycoplasmatota, and Pseudomonadota but decreased the abundance of Bacteroidota in cecal contents. It may also be associated with increased *Anaeroplasma* and *Lactobacillus*, but decreased *Bacteroides*, *Oscillospira*, *Faecalibacterium*, and *Dorea* compared to controls [65]. Studies have revealed that chickens exhibit altered feeding behavior under heat stress [67], including appetite suppression, and increased water intake [68]. These physiological changes have been linked to shifts in gut microbiota composition, suggesting that dysbiosis may be partially responsible for the detrimental effects of heat stress on growth performance. This microbial imbalance may contribute to increased intestinal permeability, as well as immunological and metabolic dysfunction [65]. Furthermore, heat stress negatively affects intestinal morphology, epithelial integrity, and mucosal immunity. Additionally, the upregulation of proinflammatory cytokines under heat stress may further contribute to microbiota dysbiosis [69–71]. In contrast, winter conditions promote the growth of potentially beneficial taxa such as members of Erysipelotrichaceae and *Phascolarctobacterium*. Erysipelotrichaceae, which includes members of the *Clostridium XVI*, produces short-chain fatty acids (SCFAs) that support host health and may help compensate for performance losses observed in the colder season [63].

Cold temperature stress can also induce long-term neuroendocrine changes in the intestinal environment, resulting in shifts in microbial composition [72]. In quail, cold stress has been shown to alter the cecal microbiota, induce oxidative stress and inflammatory damage in cecal tissues, and upregulate Hsp70 expression. This upregulation may act as a protective mechanism against cold-induced stress [73]. Seasonal effects also extend to airborne bacterial communities in poultry houses. A study investigating the concentration of airborne bacterial aerosols in a broiler house revealed that Bacillota had a higher relative abundance in spring and fall. However, Pseudomonadota were less prevalent during these seasons. *Enterococcus* and *Pseudomonas* are most abundant in winter, likely due to inadequate ventilation [74]. Microbial composition of chicken carcasses also varies with season. A previous study revealed seasonal differences in both microbial abundance and taxonomy, with *Campylobacter* being overrepresented in summer [75]. *Campylobacter* levels in chicken carcasses are positively correlated with their abundance in cecal contents [76]. While some specific pathogen linkages have been established, comprehensive studies examining the overall relationship between carcass microbiota and gut microbiota remain limited [76, 77]. Seasonal changes, especially in temperature, can induce physiological alterations in chickens that subsequently reshape the intestinal microbiota. At the same time, shifts in environmental and carcass-associated microbiota further influence the birds themselves and the entire production chain.

## Conventional farming and microbial ecology

The rising global demand for chicken meat has driven the widespread adoption of conventional poultry farming systems aimed at maximizing production efficiency and economic returns. The success of this intensive model is built on three pillars: precise genetic selection for rapid growth, optimized nutritional strategies, and strict environmental control within indoor housing systems. The following sections outline the core practices of conventional poultry farming and review recent research on how these conditions influence the composition, diversity, and function of the chicken gut microbiome.

### Characteristics and challenges of conventional farming

Conventional poultry farming is characterized by intensive production methods, where commercial broiler chickens are housed indoors with limited access to fresh air, daylight, and outdoor space [4, 78]. Flock sizes in the EU typically range from 10,000 to 40,000 birds [4]. Over the past few decades, genetic selection and optimized management practices have significantly improved growth rates, feed efficiency, and meat yield, making fast-growing broiler chickens a commercial standard. The fattening period of fast-growing broiler chickens typically lasts 28–42 d under an all-in-all-out housing system, which means that all broiler chickens in the flock are introduced and removed from a facility simultaneously [4]. Common strains used in conventional systems include the Cobb, Hubbard, and Ross strains. Between 1957 and 2005, growth rates in broiler chicken increased by more than 400%, with an average annual increase of 3.3% in live body weight at 42 d of age [4, 5]. Broiler chicken diets in conventional systems are formulated to meet nutritional requirements and support optimal growth performance. Energy, protein, and amino acid contents, and mineral contents are decisive factors influencing feed efficiency, productivity, and carcass composition [3, 79, 80]. While soybean meal remains the primary protein source, alternative plant-based protein sources such as canola and sunflower meal are also used [81, 82]. Nutrient levels are adjusted, by combining suitable components and adding synthetic amino acids and other feed supplements to the respective growth phase, with higher protein levels in early life and a shift toward increased energy content in finisher diets [3]. Despite its efficiency, conventional poultry farming faces multiple challenges. High stocking densities, a common practice used to optimize production, have been associated with adverse effects on broiler chicken health and welfare, including intestinal mucosal damage and oxidative and physiological stress. These factors can reduce growth performance, impair feed utilization, and lower carcass yield [83–85]. Moreover, intensive genetic selection for rapid growth has led to trade-offs in broiler health. Fast-growing broiler chickens are more susceptible to metabolic disorders [3, 86], have weaker immune responses [87], and often produce meat of lower quality [86, 88, 89].

### Antibiotic use and microbial ecology in conventional systems

For decades, antibiotics have been widely used in conventional poultry farms for growth promotion, therapeutic purposes, and disease prevention, ensuring flock health and performance [90, 91]. GPAs are believed to improve feed efficiency by altering the gut microbiota, suppressing pathogenic bacteria, and promoting the proliferation of beneficial microbes. In the absence of antibiotics, opportunistic pathogens may proliferate in the broiler chicken intestine, leading to intestinal inflammation, increased toxin production, decreased performance, and increased mortality rates [92, 93]. However, the routine use of antibiotics, especially as growth promoters, in conventional broiler chicken farms, has contributed to the emergence and spread of AMR, which poses a threat to animal and public health [94–96]. In response, many countries have implemented regulatory restrictions or outright bans on the use of antibiotics for growth promotion. The EU banned the use of antibiotics as growth promoters in 2006 [9], followed by similar regulatory actions in countries such as the United States, Canada, Australia, China and South Korea [97–99]. Nevertheless, the use of GPAs continues in approximately 20% of the member countries of the World Organization for Animal Health (WOAH) [100]. In regions such as parts of Southeast Asia, Latin America, and Africa, antibiotics are still legally used as growth promoters in poultry production [101]. For example, in countries such as India, Indonesia, and Bangladesh, GPAs are widely available and are routinely added to feed or water in broiler operations [102]. This continued use contributes to the persistence of AMR hotspots and presents significant challenges for global mitigation efforts.

**Fig. 2:**
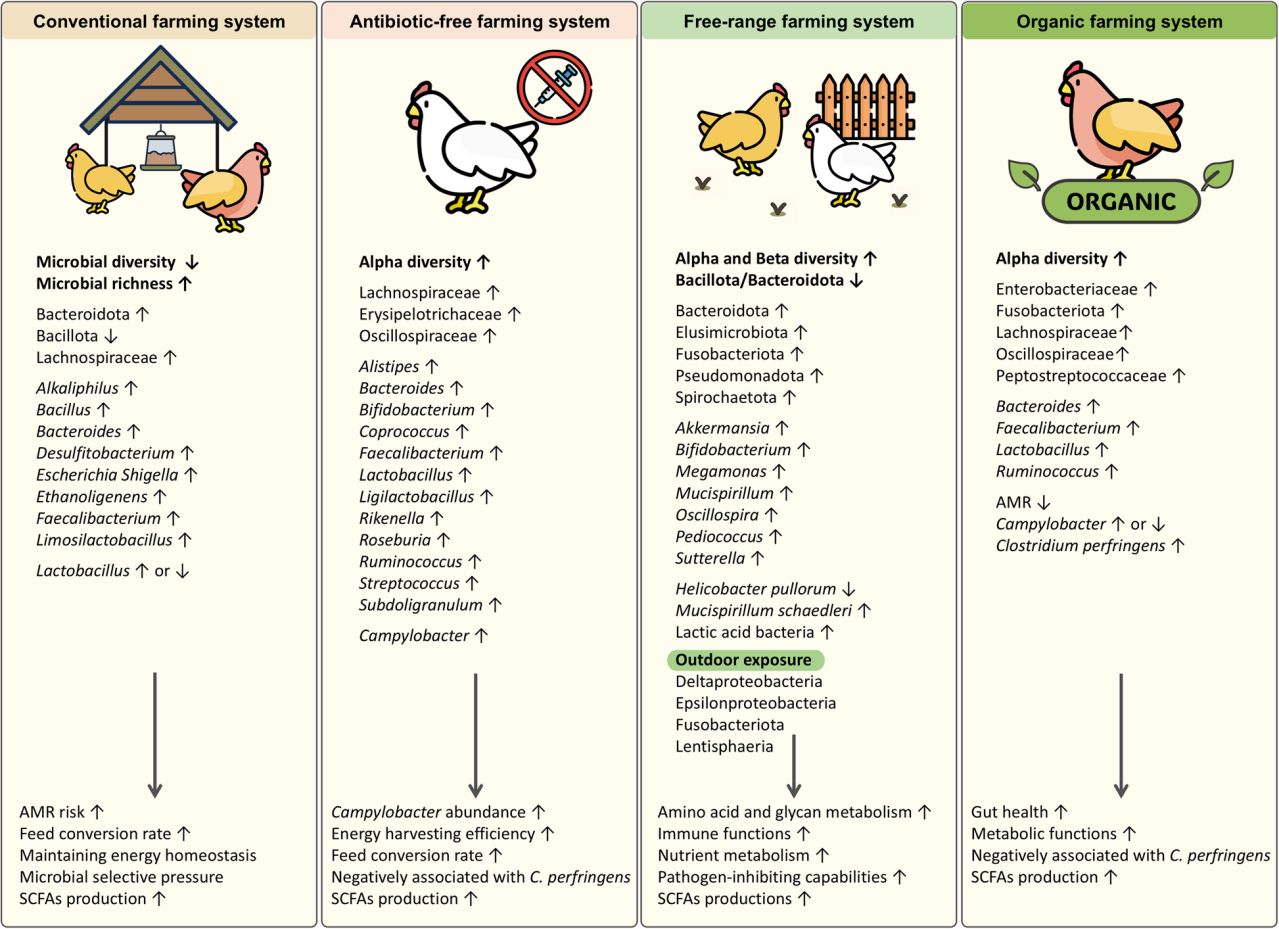
Gut microbiome composition and functionality in different chicken production systems. SCFAs: short-chain fatty acids; AMR: antimicrobial resistance; *C. perfringens*: *Clostridium perfringens*

Numerous studies have reported that antibiotic administration alters gut microbial diversity and composition (Fig. 2) [32, 103–105]. The cecal microbiota composition changes significantly following antimicrobial supplementation for growth promotion [106]. Kairmi et al. [107] reported that, compared with antibiotic-free chickens, conventionally raised chickens present greater cecal microbial diversity and richness at an early age. However, antibiotic-free chickens tend to have higher microbial diversity in later growth stages, whereas conventionally raised chickens maintain higher microbial richness. The authors attributed this pattern to antibiotic-induced reductions in microbial competition, allowing specific bacteria to proliferate [107].

Specifically, they reported that conventional chickens treated with antibiotics presented increased abundances of *Bacteroides* and *Faecalibacterium* later in life [107]. *Bacteroides* plays a key role in polysaccharide and oligosaccharide metabolism, producing SCFAs, which contribute to improved feed efficiency and microbiota homeostasis [108]. Similarly, *Faecalibacterium* offers additional benefits to the host, such as regulating lipid metabolism and maintaining glucose homeostasis in chickens. It also enhances chicken production efficiency by increasing feed conversion ratios and reducing overall feed intake [109]. Other studies have confirmed that trimethoprim-sulfamethoxazole or doxycycline alters microbial diversity throughout the GIT of chickens [104] and has long-lasting effects throughout the entire rearing period [103]. Among the antimicrobials tested, enramycin reduced microbial richness at the genus level, whereas halquinol had no significant effect on the microbial community compared with controls. Interestingly, none of the tested antimicrobial treatments altered the overall structure of cecal microbiota, suggesting that these drugs primarily impact rare or low-abundance species rather than the dominant microbial populations [106]. The impact of virginiamycin, a commonly used antibiotic, on gut microbiota remains controversial. Some studies have reported that virginiamycin reduces bacterial diversity in the chicken intestines and leads to an increased abundance of *Bacteroides* and Lachnospiraceae [110]. Virginiamycin treatment has also been shown to significantly increase *Lactobacillus* in the jejunum and *Faecalibacterium* in the cecum [105]. Another study revealed that virginiamycin supplementation led to a higher relative abundance of Bacteroidota while reducing the abundances of Bacillota and *Lactobacillus* in the cecal microbiota [111], possibly due to the inhibitory effects of virginiamycin on lactic acid bacteria (LAB) [32]. Conversely, Hodak et al. [112] reported no significant changes in cecal microbial diversity following virginiamycin administration. In a study examining six commonly used antibiotics (lincomycin hydrochloride, gentamicin sulfate, florfenicol injection, benzylpenicillin potassium, ceftiofur sodium and enrofloxacin infection), Zhan et al. [113] reported that antibiotic use disrupted the stability of the microbial community and reduced diversity. Across all the treatments, Pseudomonadota and Bacillota were the dominant phyla. Several pathogens, such as *Shigella*, *E. coli*, and *Salmonella,* have also been found in fecal samples. These discrepancies suggest that the effects of virginiamycin on the gut microbiota may be influenced by various factors, such as dosage, duration of administration, and interactions with dietary components. More targeted research is needed to clarify the specific mechanisms by which virginiamycin modulates the poultry gut microbiome. However, these microbiota differences may be influenced by various factors, including the type and dosage of antibiotic used, feed formulation, intestinal segment sampled, and the timing of sample collection. Even within the same study, microbial shifts can vary in direction across different regions of the GIT.

## Non-conventional production systems

Driven by increasing consumer concerns about antibiotic use, animal welfare, and environmental sustainability, the poultry industry has introduced a range of alternative production systems. These alternative rearing methods include antibiotic-free systems aimed at mitigating AMR; free-range systems, which provide outdoor access for chickens to enhance animal welfare; and organic farming, which prioritizes sustainable and natural rearing conditions, and includes both restrictions on the use of antimicrobials as well as provision of outdoor access. Additionally, hybrid models integrate elements from these systems to optimize both productivity and ethical considerations. The following sections discuss key microbiome-related findings in different non-conventional production systems.

### Antibiotic-free systems and microbial ecology

Antibiotic resistance is initiated through bacterial‒antibiotic interactions, where susceptible bacteria are eliminated while resistant populations thrive. The extensive use of antimicrobial agents has accelerated both the expansion and rapid emergence of drug-resistant pathogens, which can effectively transmit resistance genes across human populations, animal hosts, and environmental reservoirs [16, 114]. In response to increasing consumer concerns over antibiotic residues in poultry products and the risks of AMR, antibiotic-free poultry production has gained significant momentum in the USA and Europe [114]. This system requires optimized housing conditions, appropriate diets, and effective pathogen control strategies [16]. Maintaining gut health and a stable microbiota composition is particularly critical. Several alternative approaches have also been developed to replace antibiotics, including the use of probiotics, prebiotics, organic acids, enzymes, phytogenic additives, and essential oils [16]. Studies analyzing the core microbiota of antibiotic-free chickens revealed distinct spatial and temporal variations across different gastrointestinal sections [27, 32, 43]. *Lactobacillus* is a dominant genus at multiple sites, including the ileum, litter, and trachea, although its presence in the ceca was only detected after 28 d of age [43]. The cecal microbiota has the highest diversity, comprising core members, such as *Bacteroides*, *Faecalibacterium*, *Streptococcus*, *Oscillospira*, *Escherichia/Shigella*, *Rikenella*, and *Ruminococcus*, along with representatives of the Lachnospiraceae and Erysipelotrichaceae families. These microbial populations remain relatively stable throughout the chickens' lifespan [43]. In contrast, the microbial community in the ileum is less diverse, with specific differences at the genus level. *Ligilactobacillus* is a dominant genus in antibiotic-free chickens, whereas *Limosilactobacillus* is predominant in conventionally raised chickens [32].

### Comparison of antibiotic-free and conventional systems

Comparative analyses between antibiotic-free and conventional farming systems revealed distinct microbiome profiles (Fig. 2) [115]. Cecal microbiota in antibiotic-free systems presents significantly higher alpha diversity, an indicator of host health, particularly in older birds [107, 115]. The temporal dynamics differed between the two systems, with conventional chickens exhibiting rich but less diverse cecal microbiota than antibiotic-free chickens [107]. Some studies, however, report the opposite, suggesting that conventionally raised broilers may have more balanced microbial communities [32]. These discrepancies likely reflect the selective pressure antibiotics exert on certain taxa, which shapes microbial succession. Additionally, alpha diversity in antibiotic-free chickens is typically higher in the upper and middle sections of the GIT, but lower in the lower GIT part than in conventionally raised chickens. *Lactobacillus* predominates in antibiotic-free chickens, whereas *Escherichia/Shigella* has a higher relative abundance in the upper and middle GIT of antibiotic-treated chickens [104]. Distinct microbial signatures are associated with both production systems. Conventional farms show cecal enrichment of *Alkaliphilus*, *Desulfitobacterium*, *Bacillus*, and *Ethanoligenens*, whereas antibiotic-free farms have higher levels of beneficial butyrate-producing bacteria, including *Coprococcus*, *Roseburia*, and *Subdoligranulum* [115]. Additionally, *Bifidobacterium* and *Alistipes* are significantly more abundant in antibiotic-free chickens [32, 115]. These butyrate-producing bacteria play an important roles in maintaining gut health by enhancing intestinal barrier function, promoting intestinal mucosa repair, and reducing inflammation through multiple mechanisms [116]. Furthermore, recent metabolomic analyses have revealed that the increased presence of these SCFA-producing bacteria significantly enhances energy harvesting efficiency and improves feed conversion rates in antibiotic-free poultry systems [117, 118]. Interestingly, while the enrichment of SCFA-producing bacteria in antibiotic-free chickens is generally beneficial, certain members of Lachnospiraceae and Oscillospiraceae, known for polysaccharide fermentation, have been negatively associated with necrotic enteritis caused by *Clostridium perfringens* [107, 119]. This finding highlights the complexity of microbiota‒host interactions, and the potential trade-off of microbiome shifts in antibiotic-free systems. Another concern is the increased abundance of *Campylobacter*, a major foodborne pathogen that may carry AMR genes [120]. Interestingly, its prevalence in antibiotic-free birds has been linked to higher levels of SCFA-producing bacteria, as SCFAs serve as a carbon source for *Campylobacter*, facilitating its colonization [32, 121]. This relationship has been confirmed by studies demonstrating that microbiota-derived SCFAs act as colonization signals for *Campylobacter jejuni* [122]. Farm litter microbial communities also differ significantly between antibiotic-free and conventional production systems. Higher abundances of Clostridiaceae, Lactobacillaceae, and Corynebacteriaceae characterize antibiotic-free farm litter. Conversely, conventional farms have a predominance of Enterobacteriaceae and Bacillaceae [123]. These findings suggest that antibiotic usage patterns significantly influence the gut microbiota and the broader farm ecosystem, potentially influencing environmental reservoirs of AMR. However, taxonomic distinctions between conventional and antibiotic-free systems diminish once chickens reach slaughterhouses. Various factors, including transportation, slaughterhouse hygiene, and processing conditions, contribute to microbiota composition of chicken carcasses, reducing farms’ influence at this stage [115]. Overall, antibiotic-free chicken production systems promote a distinct gut microbiota profile with greater diversity and a higher prevalence of beneficial bacteria. However, potential risks, such as elevated *Campylobacter* levels and shifts in SCFA-producing bacteria linked to necrotic enteritis, underscore the importance of careful management. Novel feed additives such as probiotics, prebiotics, essential oils, enzymes, organic acids, and phytobiotics, are being explored as functional alternatives to antibiotics [91]. Future research should focus on refining microbial interventions to enhance gut health and maintain production efficiency while minimizing health risks in antibiotic-free poultry systems.

### Free-range farming and microbial ecology

As conventional production meets consumer demand for cheap meat, there has been growing awareness that conventional farming often overlooks animal welfare in the pursuit of higher yields. This awareness has driven the demand for free-range chickens on the market. Consumers view purchasing free-range chickens to support animal welfare as a key motivation for their choice. Additionally, the desire for better flavor, higher meat quality, and greater nutritional value encourages consumers to choose free-range chickens [15, 124, 125]. A free-range production system allows chickens to access outdoor areas during the day while typically housing them indoors at night for protection. However, regulatory definitions of free-range farming vary by region. For example, EU legislation on free-range systems covers aspects such as outdoor ground type, feed, animal density, and slaughter age [126]. In contrast, the United States primarily defines free-range systems by providing of outdoor access without strict regulations on stocking density or feed composition. Some farmers prefer slow-growing chicken genotypes for free-range systems because of their ability to obtain additional nutrients from forage and pasture and their better adaptability than compared to fast-growing lines [12, 15, 127].

Studies on slow-growing chickens, such as the Sasso-T451A line, have shown that cecal microbiota in a free-range system develops in three distinct stages throughout bird’s lifespan [25]. In the early stage of life (from hatch to two weeks), the microbiota remains immature and is characterized by the predominance of Pseudomonadota and Bacillota phyla. Within Bacillota, members of the Lachnospiraceae family are particularly abundant, whereas Pseudomonadota is primarily represented by *E. coli*. Between two and eight weeks of age, Bacillota becomes the dominant phylum [25], with Oscillospiraceae exhibiting greater abundance than Lachnospiraceae within this phylum. From eight weeks of age onward, a shift occurred as Bacteroidota partially replaced Bacillota. During this stage, Veillonellaceae accounted for nearly half of the Bacillota population, with *Megamonas* emerging as the most abundant genus, indicating that microbial composition changed before and after outdoor access. Following access to the outdoors, additional bacterial groups, such as phylum Fusobacteriota (formerly Fusobacteria), class Deltaproteobacteria*,* Epsilonproteobacteria*,* and Lentisphaeria, are detected, which are likely influenced by dietary changes, including grass consumption [25]. Other studies also reported Bacteroidota, Bacillota and Pseudomonadota as the dominant phyla in the ceca of free-range birds, including Label Hubbard hybrids [128], Dagu chickens [129] and Bermuda chickens [130]. *Clostridium* and *Ruminococcus* are the most predominant genera [128]. These birds, which are raised outdoors, may establish a more diverse microbiota earlier in life due to greater environmental exposure. A study on Cobb 308 chickens in a free-range system revealed that Bacillota remained dominant in the ileum throughout the birds’ lifespan, followed by Pseudomonadota, Bacteroidota and Mycoplasmatota (formerly Tenericutes). *Lactobacillus* was the most prevalent bacterial genus before 7 weeks of age but was replaced by *Bacteroides* in the last weeks of life [131].

### Comparison of free-range and conventional farming microbiota

Multiple studies have demonstrated distinct microbiota profiles between free-range and conventionally raised chickens [13, 132, 133]. Multiple studies have reported that free-range birds exhibit greater microbial diversity and compositional complexity; however, some investigations have yielded inconsistent or inconclusive results (Fig. 2) [25, 134]. A characteristic of free-range systems is a lower Bacillota/Bacteroidota ratio, especially toward the end of the birds’ lifespan [25, 129, 133]. These differences may be attributed to various factors, including outdoor access and the associated dietary variation in free-range systems. At the phylum level, many studies have reported increased abundances of Bacteroidota, Pseudomonadota, and Spirochaetota (formerly Spirochaetes) compared to those in conventional chickens, although the results vary depending on breed, diet, and environment [133, 135]. In one study, the phylum Methanobacteriota (formerly Euryarchaeota [136]) was found to be the only archaeal phylum detected in the ceca of older birds [25]. Fusobacteriota has been detected in various rearing systems, although multiple studies have reported higher abundance in free-range chickens compared to other management systems [25, 137, 138]. Elusimicrobiota (formerly Elusimicrobia) was also detected in free-range chicken cecal contents [25]. Distinct species identified in the cecal microbiota of free-range chickens, yet absent in conventional broiler chickens, included propionate producers such as *Megamonas* [25, 132]. Additionally, beneficial bacteria such as *Bifidobacterium*, known for their role in lactic acid production and gut health, have been reported to be enriched in free-range birds [25]. Higher levels of *Akkermansia* and the presence of *Mucispirillum* in free-range systems further suggest an increased capacity for mucus layer formation, supporting gut integrity [25, 60]. The microbiota of free-range birds has relatively low abundances of potentially harmful bacteria such as *Helicobacter pullorum*, whereas beneficial species such as *Mucispirillum schaedleri* and *Oscillospira* are enriched, potentially providing protection against pathogenic infections [139]. The distribution of LAB also differs between farming systems. Free-range chickens have relatively high levels of *Lactobacillus acidophilus*, whereas *Limosilactobacillus reuteri* and *Lactobacillus johnsonii* predominate in conventional broiler chickens [140]. System-specific species include *Lactobacillus crispatus,* which is exclusive to conventional broiler chickens, and *Limosilactobacillus vaginalis* and *Ligilactobacillus agilis,* which are unique to free-range systems [140]. One study reported that *Pediococcus* is approximately ten times more prevalent in free-range chickens and is detected mainly in young chicks [25]. These LAB strains in free-range chickens have strong pathogen-inhibiting capabilities, suggesting their potential as natural alternatives to conventional antibiotic growth promoters [140]. Moreover, while *Campylobacter* appears at later stages in free-range chickens, *Sutterella* remains the dominant genus in this group, potentially acting as a direct competitor to *Campylobacter* within the intestinal ecosystem, thereby limiting its colonization [25]. The distinct microbial compositions in free-range systems also translate into functional advantages. Free-range chickens show an increased capacity for amino acid and glycan metabolism [129]. The gut microbiota of these individuals expresses relatively high levels of genes involved in acetate production, which facilitates the conversion of butyrate, a short-chain fatty acid that serves as an essential energy source for the host [133]. These metabolic differences suggest that the free-range environment may promote more diverse and beneficial microbial functions. Shi et al. [141] reported that functional gene classification in free-range systems significantly increased energy production, carbohydrate transport, and amino acid transport in Lohmann hens. The Kyoto Encyclopedia of Genes and Genomes (KEGG) pathway analysis revealed significant differences in functional gene expression between cage-reared and free-range chickens, particularly within the peroxisome proliferator-activated receptor (PPAR) signaling pathway, which regulates inflammation, energy homeostasis, and intestinal homeostasis [132, 142, 143]. Although results from studies on free-range systems are very diverse, the collective evidence indicates that such systems do indeed alter the gut microbiota composition and function. These findings suggest that free-range rearing systems may support more robust metabolic and developmental processes in the intestinal tissue. However, researchers have also reported the upregulation of microbial pathways associated with human diseases, suggesting a potential increased risk of parasite exposure due to outdoor access [144]. While the free-range system supports a diverse and functionally beneficial microbiota, the presence of certain pathogens and the expression of microbial pathways linked to disease warrant further investigation.

### Organic farming and microbial ecology

Organic poultry farming is subject to distinct regulatory frameworks that vary across countries. In Europe, it is regulated under EU Regulation 2018/848, which prioritizes high animal welfare standards and promotes overall poultry health and well-being. The regulation mandates the use of slow-growing strains, imposes lower stocking densities per square meter, and requires outdoor access for at least one-third of their lifespan. Feed must be certified organic and, whenever possible, should be sourced directly from the farm [145]. The use of free amino acids is not allowed, and birds must be given free access to roughage and water. Medication use is strictly regulated. Preventive treatments, including antibiotics and artificial growth promoters, are strictly prohibited. If therapeutic use of antimicrobials is indicated, the withdrawal period is extended to twice the normal length and a minimum of 48 h. Birds that receive more than one antibiotic treatment per fattening cycle cannot be marketed as organic. Moreover, organic husbandry practices aim at enabling animals to engage in their natural behaviors, including movement, social, and exploratory behavior [145].

### Gut microbiota in organic systems

Saati-Santamaría et al. [146] reported that several bacterial taxa were identified in the feces of organically raised chickens, with *Lactobacillus*, *Faecalibacterium*, *Ruminococcus*, *Bacteroides*, Lachnospiraceae, Peptostreptococcaceae, Oscillospiraceae, and Enterobacteriaceae being the predominant groups. *Lactobacillus* presented the highest relative abundance, accounting for up to 80%, compared with less than 60% in conventionally raised and fast-growing chickens. During the first week after hatching, chickens presented a high abundance of *Lactobacillus* accompanied by enriched metabolic functions, including propanoate, pyruvate, fructose, and mannose metabolism, as well as fatty acid and lipid biosynthesis. Approximately half of these metabolic functions are attributed to *Lactobacillus*, suggesting that these bacteria play a critical roles in fructose and mannose metabolism during early development [146]. By the second week of age, *Bacteroides* became more dominant in organic rearing systems. This genus is known for fiber fermentation and may participate in cross-feeding interactions with *Lactobacillus*, leading to the production of SCFAs, which provide gut health benefits and help control pathogens such as *C. perfringens* [147].

### Comparison with conventional systems

Several studies have reported notable differences between organic and conventional chickens (Fig. 2). One study using cultivation revealed that *Clostridium perfringens* levels were significantly higher in organically raised chickens. This was attributed to the absence of salinomycin, an antibiotic widely used in conventional systems to suppress *C. perfringens* proliferation, which is not used in organic farming. Additionally, *Lactobacillus* abundance also differed between the two production systems [148]. Another study comparing fecal microbiota from layers reared in an organic antibiotic-free system versus those reared in a conventional-chlortetracycline-treated system [90] reported higher relative abundances of Fusobacteriota, *Lactobacillus* and *Clostridium* in the organic system. Interestingly, fewer AMR genes were detected in conventional chickens. A study using alpha diversity metrics have reported higher microbial diversity in organic flocks across different age groups [21]. However, research on the microbiome of organic rearing systems remains limited, with most studies focusing on pathogen prevalence, particularly antimicrobial-resistant bacteria, rather than comprehensive taxonomic or functional analysis.

### Pathogen prevalence and antibiotic resistance in organic systems

Studies investigating *Campylobacter* prevalence in organic poultry systems have yielded mixed results. One study reported that organic chickens had a lower *Campylobacter* prevalence and smaller bacterial populations in both fecal samples and earlier slaughterhouse processing stages. This was observed despite the increased exposure of organic chickens to wildlife reservoirs and environmental contamination, which would typically increase infection risk [149]. Conversely, another study reported a higher prevalence of *Campylobacter* in organic farms but with lower levels of AMR [150]. A similar trend was observed in turkey farming, where *Campylobacter* species were highly prevalent in both conventional and organic systems, yet AMR rates were significantly lower in organic farms [151]. These findings may be attributed to multiple factors, such as breed, housing, feed, climate, and age, which influence microbiota. Regional variations in organic regulations may also contribute to diverse results. Identifying a single determinant of pathogen prevalence remains challenging. Owing to this complexity, identifying a single determinant responsible for *Campylobacter* prevalence or AMR patterns in organic systems remains difficult. Further comparative studies, with standardized metadata and controlled experimental designs, are needed to clarify the ecological and epidemiological implications of organic rearing practices on zoonotic pathogen carriage and resistance. The microbiota associated with chicken meat is influenced by multiple factors throughout the production chain, including rearing conditions, transport, slaughter practices, and processing hygiene. These factors can shape the microbial communities present on carcasses and ultimately impact meat quality, safety, and shelf-life. A recent study revealed that chickens from different rearing systems exhibit distinct fecal microbiota, but these differences diminish significantly once the birds reach the slaughterhouse. Factors such as transportation stress, feed withdrawal, lairage environment, and slaughter line conditions homogenize microbial communities across carcasses [115]. Despite the lower overall prevalence, tetracycline-resistant *Campylobacter* strains, as well as erythromycin-resistant strains, are more frequently found in organic chicken carcasses than in conventionally raised ones, [152]. These findings suggest that organic farming may not necessarily reduce the levels of antimicrobial-resistant *Campylobacter* strains [153]. Previous studies revealed no significant differences in microbial diversity and abundance across production systems [154, 155]. Similarly, culture-based assessments comparing conventional and free-range chicken meat yielded no significant differences [156]. Some researchers suggest that the microbiota of chicken meat is shaped more by processing conditions than by the production system itself [155]. Owing to the limited number of studies focusing on the gut microbiome of organic chickens, research examining chicken carcasses and meat microbiota can provide alternative insights into how different production systems influence the overall microbial ecology of poultry.

## Metadata gaps in poultry microbiome research

During our investigation into the relationship between microbiome composition and chicken farming systems, we observed substantial heterogeneity and limited reproducibility among the reported findings. Although microbiome-based approaches are increasingly applied in poultry research, many published studies continue to show considerable variation in experimental design and frequently lack detailed metadata. Beyond commonly reported factors such as age, breed, season, and rearing system, numerous additional variables, including GIT region, sample type, maternal influences, sex, diet, hygiene practices, vaccination status, and environmental conditions, can strongly influence microbial composition and function [20, 157].

Moreover, methodological aspects such as DNA extraction, sequencing platforms, reference database selection, sample collection procedures, storage conditions, and bioinformatics pipelines further shape microbiome outcomes [158]. Consequently, findings from poultry microbiota studies are often difficult to reproduce or compare across studies. The frequent underreporting of influential variables further complicates the interpretation of microbiome data and practical context. One of the most persistent challenges lies in the inconsistent and often ambiguous terminology used to describe farming systems. Additionally, the frequent omission or vague reporting of chicken diet composition complicates efforts to collect, integrate, and compare microbiome data across studies. As scientists have already suggested that poultry microbiome research requires a standardized protocol [158]. We propose that future publications in this research area adopt more standardized and detailed descriptions of farming practices. Studies should consistently report key aspects of husbandry management, including the type of chicken production, such as conventional, organic, free-range, or antibiotic-free systems, flock size, stocking density, and the provision of outdoor access, including the age at which birds are first granted access. In addition, information should be provided on the use of medication, particularly antibiotics and their alternatives, as well as feed composition, including ingredient sourcing and processing, and any supplementary additives such as probiotics, prebiotics, or enzymes.

Given the well-established influence of these variables on gut microbial communities, enhanced transparency in methodological reporting is essential. The provision of detailed metadata will not only improve the reproducibility of findings but also facilitate more precise, systematic comparisons across studies. This, in turn, will support more reliable interpretation of microbiota patterns and their associations with specific farming practices, ultimately contributing to the development of microbiome-informed strategies for sustainable poultry production.

## Conclusion

The gut microbiota represents a critical determinant of poultry performance, influencing nutrient metabolism, immune competence, disease resistance, and overall productivity. As poultry production systems evolve to reduce reliance on antibiotics and meet rising standards for animal welfare and sustainability, a deeper understanding of how rearing practices shape gut microbial communities is essential. Insights into these microbiota–host–environment interactions can inform the development of targeted nutritional and management strategies to optimize bird health and production efficiency.

This review examines the impact of age, genetic line, and seasonal factors on the diversity and structure of the chicken gut microbiota. Age-related microbial succession, selective breeding, and seasonal changes collectively shape the gut environment and microbial communities, with downstream effects on bird physiology and production outcomes. These factors impact microbial ecosystems by modulating host traits, including immune response, growth performance, and nutrient absorption capacity.

Additionally, this review also demonstrates that production systems, ranging from conventional to antibiotic-free, free-range, and organic systems, differ markedly in their effects on microbiota composition, diversity, and functional potential. In conventional systems, the use of antibiotics can substantially influence the gut microbiota by modulating its composition and controlling potential pathogens, thereby contributing to microbial profiles that differ markedly from those observed in alternative rearing systems. Non-conventional systems often promote higher microbial diversity and the enrichment of beneficial taxa such as SCFAs producers and LAB. However, they may also increase exposure to environmental pathogens, highlighting the need for targeted microbial management strategies. To advance the field, future research should prioritize integrative approaches that combine metagenomics, metabolomics, and other high-resolution omics technologies to clarify the functional consequences of microbiota shifts across production systems. These insights will support the development of next-generation feed additives, including probiotics, prebiotics, enzymes, and phytogenics, that strategically modulate the gut microbiome to improve animal health and productivity. Given the high degree of heterogeneity among existing study results, we advocate for the future standardization of research protocols in poultry microbiome studies to enhance reproducibility and comparability across investigations. In parallel, the adoption of standardized metadata reporting frameworks will enhance data reproducibility and enable more robust comparisons across studies. Together, these efforts will contribute to the implementation of microbiome-informed strategies for efficient, resilient, and sustainable poultry production.

## Data Availability

Not applicable.

## Abbreviations

AMR: Antimicrobial resistance
EU: European Union
GIT: Gastrointestinal tract
GPAs: Growth-promoting antibiotics
KEGG: Kyoto Encyclopedia of Genes and Genomes
LAB: Lactic acid bacteria
PPAR: Peroxisome proliferator-activated receptor
SCFA: Short-chain fatty acid
